# Interplay among SNAIL Transcription Factor, MicroRNAs, Long Non-Coding RNAs, and Circular RNAs in the Regulation of Tumor Growth and Metastasis

**DOI:** 10.3390/cancers12010209

**Published:** 2020-01-14

**Authors:** Klaudia Skrzypek, Marcin Majka

**Affiliations:** Jagiellonian University Medical College, Faculty of Medicine, Institute of Pediatrics, Department of Transplantation, Wielicka 265, 30-663 Cracow, Poland

**Keywords:** tumor, metastasis, microRNA, SNAIL (SNAI1) transcription factor, epithelial to mesenchymal transition (EMT), long non-coding RNAs (lncRNAs), circular RNAs

## Abstract

SNAIL (SNAI1) is a zinc finger transcription factor that binds to E-box sequences and regulates the expression of genes. It usually acts as a gene repressor, but it may also activate the expression of genes. SNAIL plays a key role in the regulation of epithelial to mesenchymal transition, which is the main mechanism responsible for the progression and metastasis of epithelial tumors. Nevertheless, it also regulates different processes that are responsible for tumor growth, such as the activity of cancer stem cells, the control of cell metabolism, and the regulation of differentiation. Different proteins and microRNAs may regulate the SNAIL level, and SNAIL may be an important regulator of microRNA expression as well. The interplay among SNAIL, microRNAs, long non-coding RNAs, and circular RNAs is a key event in the regulation of tumor growth and metastasis. This review for the first time discusses different types of regulation between SNAIL and non-coding RNAs with a focus on feedback loops and the role of competitive RNA. Understanding these mechanisms may help develop novel therapeutic strategies against cancer based on microRNAs.

## 1. Introduction: Background of SNAIL Transcription Factor

SNAIL is a member of the group of conservative zinc finger transcription factors. It was first described in *Drosophila melanogaster* as an essential factor for the mesoderm formation [1]. Subsequently, its homologues have been described in many species, including humans. The SNAIL family consists of three members: SNAIL (SNAI1), SLUG (SNAI2), and SMUG (SNAI3) [2]. The SNAIL protein contains C-terminal zinc finger domains that are responsible for DNA binding, the N-terminal SNAG domain responsible for interaction with several co-repressors or epigenetic remodeling complexes, the serine-rich domain (SRD) regulating ubiquitination and proteasome degradation, and the nuclear export sequence (NES) that controls the protein stability and subcellular localization [3].

### 1.1. SNAIL Expression and Regulation

SNAIL expression may be regulated by many signaling pathways. At the transcriptional level, SNAIL is regulated by multiple growth factors and signaling molecules that are responsible for the subsequent regulation of the SNAIL promoter, including transforming growth factor β (TGF-β), fibroblast growth factor 2 (FGF2), epidermal growth factor (EGF), Harvey rat sarcoma viral oncogene homolog (H-ras), Akt kinase-transforming protein (v-Akt), and nuclear factor kappa-light-chain-enhancer of activated B cells/protein 65 (NF-κB/p65) [4,5]. Post-translational modifications, such as phosphorylation, ubiquitination, and lysine oxidation also regulate SNAIL level. Glycogen synthase kinase 3 beta (GSK-3β) phosphorylates SNAIL at two consensus motifs. Phosphorylation of the first motif regulates ubiquitination and degradation in the proteasome, whereas phosphorylation of the second motif regulates its subcellular localization [6]. Lysyl oxidase-like 2 (LOXL2) enzyme interaction regulates SNAIL stability [7] by interfering with FBXL14 binding SNAIL. FBXL14 (F-box and leucine-rich repeat protein 14) is a ubiquitin ligase that targets both phosphorylated and unphosphorylated SNAIL for proteasome degradation [8]. SNAIL can also be stabilized by hyperglycemia-regulated O-linked β-N-acetylglucosamine (O-GlcNAc) modification of serine [9]. Moreover, SNAIL can be stabilized by NF-κB, which induces COP9 signalosome 2 (CSN2), which, in turn, blocks the ubiquitination and degradation of SNAIL [10]. The phosphorylation of SNAIL may result in an increased retention of the protein in the nucleus. That mechanism of action was described for p21-activated kinase (PAK1), which phosphorylates SNAIL at Ser 246 [11].

### 1.2. Different Pathways Regulated by SNAIL

SNAIL plays an important role in the regulation of epithelial to mesenchymal transition in embryo development: gastrulation and mesoderm formation [2]. However, molecular mechanisms of certain pathological stages resemble those observed in physiological process. One of them is epithelial to mesenchymal transition (EMT) during cancer progression. It is the main mechanism responsible for the invasiveness and metastasis of neoplasm at the advanced stages [12]. SNAIL exerts its effects by decreasing the expression of E-cadherin by binding to its promoter [13]. Nevertheless, SNAIL is a transcriptional repressor, which binds to regulatory regions and promoters containing sequences called E-boxes, and thereby it regulates the expression of many different genes and in this way, it may also regulate EMT. The SNAIL family contains a highly conserved region of four to six zinc fingers that allows them to interact with those E-box sequences (CANNTG). Since these sequences are also recognized by transcription factors from the basic helix-loop-helix (bHLH) family, the role of SNAIL factors is mainly focused on transcription repression by excluding these proteins from their binding sites [2]. SNAIL is capable of interacting with HDAC1/2 histone deacetylase, which causes a local modification of the chromatin structure and blocks the expression of E-cadherin, the loss of which is a marker of epithelial–mesenchymal transition (EMT) [13]. As E-box sequences are present in the promoters of many different genes, in the literature, SNAIL is described as a regulator of many genes important in tumorigenesis, such as cyclin D2, proliferating cell nuclear antigen (PCNA), prostaglandin dehydrogenase, ATPase1, etc. [12]. SNAIL turned out to be also a direct regulator of not only EMT in tumor progression, but also of myogenic differentiation. The binding of SNAIL to E-box sequences in the myogenic factor 5 (MYF5) promoter and recruiting histone deacetylases (HDACs) was described in the regulation of rhabdomyosarcoma development [14]. Another example of the non-canonical actions of SNAIL is the regulation of myoblast determination protein 1 (MyoD) function in myogenic differentiation by the competitive binding of SNAIL to its regulatory sequences [15]. Nevertheless, SNAIL is not only described as a transcriptional repressor, but also as the transcriptional activator. For example, SNAIL induces the expression of mesenchymal genes, such as vimentin, fibronectin, matrix metalloproteinases MMP-2, and MMP-9. In that way, it further facilitates the increased motility of cells [16].

What is more, the recent data demonstrated the mechanism of self-regulation by members of the SNAIL family: the SNAIL-binding site is present in the *SNAIL* promoter (negative feedback) [17], and avian Slug can self-activate during the neural crest development [18]. Moreover, in ovarian cancer cells, SNAIL binds to two E-box sequences in *SLUG* promoter and represses SLUG, which is predominantly mediated through the recruitment of the HDACs [19].

SNAIL plays a role in many physiological and pathological processes, such as chronic inflammation, fibrosis, EMT induction, the regulation of cancer stem cells, the control of cell metabolism, the suppression of estrogen receptor signaling, and in particular the development and metastasis of tumors [3]. Currently, many research papers focus not only on interaction between SNAIL and different genes, but also on the interplay between SNAIL and non-coding RNAs, such as microRNAs, long non-coding RNAs, and circular RNAs [20]. In this review, we discuss recent advances in those fields. We present bidirectional crosstalk between SNAIL and non-coding RNAs with implications of these new findings on tumor progression, which may help develop novel therapeutic strategies in future.

## 2. Non-Coding RNAs as Regulators of Tumor Progression

**Figure 1:**
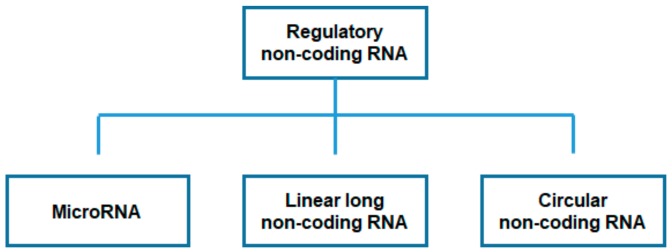
Scheme presenting the selected regulatory non-coding RNAs.

Non-coding RNAs (ncRNAs) are a class of RNA transcripts that do not encode proteins, but they may play a role in the regulation of gene expression at transcriptional, translational, and post-translational levels. Among regulatory ncRNAs, long non-coding RNAs, small RNAs, and circular RNAs may be distinguished [21] (Figure 1), and they are described in this review.

Long non-coding RNAs (lncRNAs) are RNA transcripts with a length greater than 200 nucleotides. They can regulate gene expressions and functions. Therefore, they are involved in the pathogenesis of many diseases, including cancer. Nevertheless, there are papers revealing that some lncRNAs contain cryptic open reading frames (ORFs), which may blur the distinction between protein-coding and non-coding transcripts [22]. lncRNAs can originate from their own promoters or from the promoters shared with other coding or non-coding genes, or from enhancer sequences. lncRNAs are usually transcribed by RNA polymerase II or RNA polymerase III. They are often 5′-capped, spliced, and polyadenylated, but they are usually shorter than mRNAs [23,24]. lncRNAs may be co-regulated with mRNAs in expression networks. lncRNAs may also be generated from the divergent transcription from shared protein-coding gene promoters. Divergent transcription generates the sense (mRNA) and anti-sense RNAs [24,25]. lncRNA promoters are usually evolutionarily conserved and tightly regulated, and they are prone to epigenetic modification [23]. lncRNAs may also be processed in different ways than mRNAs, such as RNase P-processed 3′ maturation, which was shown for MALAT1 (metastasis associated lung adenocarcinoma transcript 1) [24]. DICER1 endonuclease is an important factor in both the biogenesis of miRNAs that may also act as a downstream activator of many lncRNAs [26]. What is also interesting is that few miRNAs are derived from lncRNA exons [27]. lncRNAs participate in and modulate the various cellular processes, such as cellular transcription, the modulation of chromatin structure, DNA methylation, or histone modification. They may act as a sponge for microRNAs and as a competing endogenous RNAs (ceRNAs) [28].

Circular RNA (circRNA) is a type of single-stranded RNA that forms a covalently closed continuous loop that is insensitive to ribonucleases. circRNAs are formed by exon skipping or back-splicing events. circRNAs are produced by nonsequential exon-exon back-splicing, which results in a chemically circularized transcript in which 3′ sequences are spliced upstream of 5′ sequences, and they have special 5′ and 3′-end processing [24]. Alternative splicing factor quaking is a regulator of that circularization during EMT [29]. There is also a class of circular intronic lncRNA (ciRNAs) that are generated from stabilized introns after canonical splicing. They display regulatory functions, mostly at their transcription sites [30]. There are also exon–intron circRNAs (elciRNAs) that represent a class of circular RNAs that retain unspliced introns. Their role involves induction of the transcription of their parental genes via interaction with polymerase II and U1 snRNP (small nuclear ribonucleoprotein) [31]. circRNAs are closely associated with tumor metastasis and patient prognosis, because they are differentially expressed in different tumor types. They may act as a microRNA sponge and interact with proteins [32]. Nevertheless, recent research papers provide initial evidence for certain endogenous circRNAs coding for proteins [33].

MicroRNAs (miRNAs) are a class of approximately 22 nucleotides small non-coding RNAs. They can regulate the expression of genes and translation of proteins by interfering with ribosomal machinery. They commonly target the 3′ untranslated regions (3′ UTRs) of mRNAs and in that way decrease their stability and suppress translation. Nevertheless, they can also activate other genes [34,35]. Genes highly and constitutively expressed usually display shorter 3′ UTR sites and in consequence only a few binding sites for miRNAs. Accordingly, genes potently regulated during development display multiple binding sites for miRNAs [36].

miRNAs can be expressed at high levels (even up to tens of thousands of copies per cell), and they act as important regulatory factors, controlling hundreds of mRNA targets [37]. Animal miRNAs target the 3′ UTRs of different mRNAs by seed sequence complementarity. They usually repress translation more often than they cleave mRNA [35,38,39].

miRNAs are located in introns of coding genes, in exons, or in non-protein coding DNA regions. miRNAs have their own promoters, and they are independently expressed. Some of them are also organized in clusters sharing the same transcriptional regulation. miRNAs can arise from spliced introns, which are often termed miRtrons, or their own promoter, driving the expression of a single miRNA or polycistron yielding multiple pre-miRNA stem loops [40]. Nevertheless, miRNA transcription may also be dependent on the host gene. Intronic miRNAs can be expressed together with their host gene mRNA, and they can be derived from a common transcript [41]. Many non-canonical miRNA biogenesis pathways have also been characterized [42].

miRNAs are transcribed by polymerase II, sometimes as polycistronic transcripts. miRNA stem loops are excised from the primary transcripts (pri-miRNA) in the nucleus by endoribonuclease Drosha, acting together with DGCR8. Then, the excised 70–100 nt hairpin called pre-miRNA is actively transported from the nucleus to cytoplasm in a GTP (guanosine-5′-triphosphate)-dependent manner. The export is mediated by exportin 5 and Ran GTPase. Subsequently in the cytoplasm, the pre-miRNA is cleaved by Dicer endonuclease, giving the mature miRNA—a base-paired double-stranded processing intermediate with a 2 nt 3′ overhang. Two strands are generated. Then, one strand of the duplex is incorporated into RNA-induced silencing complexes (RISC) with the Argonaute protein, which is capable of endonucleolytic cleavage [42,43]. The translational repression is characterized by low miRNA–target complementarity, whereas mRNA degradation requires a high miRNA–target complementarity [44].

Alterations of miRNAs expression in various cancers have been described in the literature. Firstly, in 2002, they were shown in the most common form of adult leukemia, B cell chronic lymphocytic leukemia [45] and then in 2003 in colorectal cancers [46]. It soon turned out that miRNAs can be differentially expressed in different tumor types as either benign or malignant, and they can also act as biomarkers [47].

Global miRNA downregulation is a common trait of many tumors [48,49]. Accordingly, the diminished expression of miRNA processing factors is also associated with the poor prognosis of different cancer types [50].

What is more, some miRNAs’ loci often display genomic instability in cancer, and they are located in cancer-associated genomic regions or in fragile sites. It was also demonstrated that several miRNAs located in deleted regions are expressed at low levels in cancer [51].

Cancer cells can also escape from miRNA regulation by the production of mRNAs with shortened 3′UTR and fewer miRNA target sites. This global switch of the use of miRNA-mediated gene regulation is associated with an increased proliferation or cellular transformation [50]. These findings are consistent with the widespread decrease of miRNAs in cancer [48,49].

Some miRNAs can behave as oncogenes favoring tumorigenesis. They are called oncomirs. They can reduce the levels of proteins blocking proliferation and migration and activating apoptosis. Many miRNAs were identified as oncomirs in different types of tumors. For example, members encoded by the miR-17-92 cluster were previously associated with carcinogenesis and usually display increased expression in tumors, including lung cancer [52,53].

On the other hand, tumor-suppressive miRNAs can inhibit cancer development. Their inactivation in tumors is followed by the accumulation of proteins stimulating proliferation and migration and decreasing apoptosis. For example, miR-181a and miR-181b were described to act as tumor suppressors in glioma [54] and miR-181a in non-small cell lung carcinoma [55]. Interestingly, plenty of miRNAs may behave oppositely in different types of tumors. For example, miR-34c can exert tumor-suppressive functions in prostate cancer [56], but in lung adenocarcinoma with different oncogenic mutations, it was reported to be upregulated [57].

miRNAs can affect tumor progression also by modulation of the development of new blood vessels. miRNAs promoting angiogenesis are called angiomirs, and they can target genes that are important in angiogenic processes [58].

Currently, miRNAs’ role in the regulation of epithelial to mesenchymal transition has been widely described in the literature [59]. Since SNAIL is one of the crucial factors regulating EMT, the interplay between SNAIL and miRNAs may be a key factor in the regulation of tumor progression.

## 3. MicroRNAs Regulating SNAIL

### 3.1. MicroRNAs Directly Targeting SNAIL

**Figure 2:**
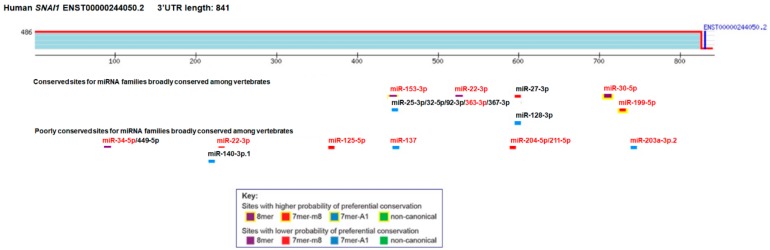
MicroRNAs targeting the 3′ untranslated (3′UTR) region of *SNAIL* from bioinformatical analysis using TargetScanHuman 7.1 (access: 22 October 2019). Experimental evidence for direct binding to *SNAIL* 3′UTR was shown in the literature for miR-153, miR-22, miR-30, miR-363, miR-199, miR-34, miR-22, miR-137, miR-203, miR-125, miR-211, and miR-203 (marked in red), which is described in the text below.

MiRNAs can act as regulators of SNAIL expression by binding to the 3′UTR of *SNAIL*. Bioinformatical analysis using TargetScanHuman 7.1 [60] revealed several binding sites for different miRNAs in this region in human cells (Figure 2), and most of them have been already verified in the literature. For example, the *SNAIL* 3′UTR was shown to function as a sponge for multiple migration and invasion-related miRNA candidates including miR-153, miR-199a-5p, miR-203, miR-204, miR-22, miR-34a and miR-34c [61].

Several miRNAs were experimentally validated to target *SNAIL* 3′UTR, and subsequently, their role was described in different tumor types. One of the crucial regulators of SNAIL expression widely described in the literature is the miR-30 family. Members of this family target the 3′UTR of *SNAIL* mRNA in non-small cell lung carcinoma [62], breast cancer [63], pancreatic cancer stem cells [64], melanoma [65], esophageal squamous cell carcinoma [66], rhabdomyosarcoma [14], or in hepatocytes [67,68]. This inhibition usually regulates EMT in epithelial tumor types, but in mesenchymal tumors, such as rhabdomyosarcoma, it may be responsible for non-canonical SNAIL action [14]; it might also be important in different processes, such as atherosclerosis [69]. Moreover, miR-30a was also shown to regulate not only SNAIL but also SLUG in breast cancer to suppress EMT and metastasis [70].

SNAIL-dependent EMT in cancer has also been demonstrated to be regulated by p53 and miR-34 axis. In the absence of wild-type p53 function, SNAIL-dependent EMT is activated in colon, breast, lung carcinoma cells [71], and ovarian cancer [72] as a consequence of a decrease in miR-34 levels. A conserved miR-34a/b/c seed-matching sequence was detected in the *SNAIL* 3′-UTR. Moreover, there is a double-negative feedback loop in the regulation of EMT formed by miR-34 and SNAIL [73]. Luciferase reporter assays revealed that in pancreatic cancer, miR-34a targets both *SNAIL* and *NOTCH1* to inhibit pancreatic cancer progression through the regulation of EMT and NOTCH signaling pathways [74].

Another example of miRNA that is described as a direct regulator of SNAIL expression in plenty tumor types is miR-153. The downregulation of SNAIL by miR-153 suppresses human laryngeal squamous cell carcinoma migration and invasion [75], melanoma cells proliferation and invasion [76], esophageal squamous cell carcinoma progression [77], and gastric cancer metastasis [78]; regulates EMT in hepatocellular carcinoma [79]; and diminishes pancreatic ductal adenocarcinoma migration and invasion with miR-153 serving as a prognostic marker [80].

MiR-22 was demonstrated to target *SNAIL* and thereby inhibit tumor cell EMT and invasion in lung [81] and bladder cancer [82], in melanoma [83] and gastric cancer [84]. In bladder cancer, it inhibits both SNAIL and MAPK1 (mitogen-activated protein kinase 1) /SLUG/vimentin feedback loop [82], whereas in melanoma and gastric cancer it acts as a tumor suppressor by targeting both SNAIL and MMP14 [83,84].

*SNAIL* was found to be a target of multiple miRNAs in different tumor types. *SNAIL* was targeted in breast cancer by miR-125b [85], miR-203 [86], miR-410-3p [87], and miR-182 [88]; in gastric cancer by miR-491-5p [89] and miR-204 [90]; in lung cancer by miR-199a [91] and miR-940 [92]; in papillary thyroid carcinoma by miR-199a [93]; in ovarian cancer by miR-137 [72] and miR-363 [94]; in hepatocellular carcinoma by miR-122 [95] and miR-502-5p [96]; in prostate cancer by miR-486-5p [97]; and in renal cancer by miR-211-5p [98]. What is more, besides tumorigenesis, SNAIL is also regulated in different processes by miRNAs. For example, miR-133 promotes cardiac reprogramming by the direct repression of SNAIL and silencing fibroblast signatures [99], whereas miR-130b directly targets SNAIL in the regulation of diabetic nephropathy [100]. The results described above are summarized in Table 1.

### 3.2. Other Examples of SNAIL Regulation by MicroRNAs

The indirect regulation of SNAIL involves several different mechanisms. One of the examples is inhibition of the GSK-3β (glycogen synthase kinase 3 beta) pathway. miR-148a binds to the 3′-UTR region of *MET*, which results in the attenuation of its downstream signaling, inhibition of AKT-Ser473 and GSK-3β phosphorylation, and in consequence reduced accumulation of SNAIL in the nucleus, the inhibition of EMT, and the metastasis of hepatoma cells [101]. In lung cancer cells, miR-126 affects the PI3K/AKT/SNAIL (phosphatidylinositol 3-kinase/protein kinase B/SNAIL) signaling pathway to regulate EMT [102]. A similar mechanism was described for miR-215 in papillary thyroid cancer [103]. In thyroid carcinoma, miR-101 targets the CXCL12 (C-X-C motif chemokine ligand 12, stromal cell-derived factor 1)-mediated AKT and SNAIL signaling pathways to inhibit invasion and the EMT-associated signaling pathways [104]. On the other hand, in hepatocellular carcinoma, miR-1306-3p targets FBXL5 to suppress SNAIL degradation and promote metastasis [105]. The SNAIL level can also be stabilized by miRNAs. miR-181b-3p promotes EMT in breast cancer cells through SNAIL stabilization by directly targeting the YWHAG protein [106]. In breast cancer cells, miR-5003-3p promotes EMT also through SNAIL stabilization via MDM2 and the direct targeting of E-cadherin [107]. In melanoma growth and metastasis, miR-9 is described as a downregulator of NF-κB1-SNAIL pathway [108]. The results described above are summarized in Table 2.

Sometimes, the research data demonstrate the regulation of SNAIL expression by miRNAs, but it is not described if the regulation is direct or indirect. There are also several other examples of miRNAs regulating the SNAIL level. In ovarian cancer, miR-16 is associated with the downregulation of mesenchymal markers, such as SNAIL, SLUG, and vimentin [109]. In Wilms’ tumor cells, miR-483-3p regulates EMT by the modulation of E-cadherin, N-cadherin, SNAIL, and vimentin expression [110]. In osteosarcoma, the downregulation of miR-145 promotes EMT by regulation of the SNAIL level [111]. In rhabdomyosarcoma, miR-410-3p inhibits tumor growth and progression by inhibition of the expression of SNAIL, SLUG, N-cadherin, and Bcl-2 [112]. However, miR-410-3p was shown previously in different tumor types to directly target SNAIL [87].

The miRNAs–SNAIL axis may regulate not only EMT, but also the activity of cancer stem cells. miR-210 induced by a hypoxic microenvironment favored breast cancer stem cells’ metastasis, proliferation, and self-renewal by targeting E-cadherin and the upregulation of SNAIL [113]. Another example is miR-146a, which directs the symmetric division of SNAIL-dominant colorectal cancer stem cells [114].

### 3.3. Regulation of SLUG Expression by MicroRNAs

MiRNAs can regulate not only SNAIL, but also SLUG, which is another important factor from the SNAIL family. Some miRNAs can regulate both factors. Among them are miR-30a [70], miR-122 [95], miR-182 [115], and miR-203 [115] and miR-204 [116]. *SLUG* is targeted in in oral squamous cell carcinoma by miR-204 [116]; glioblastoma by miR-203 [117]; in lung cancer by miR-1 [118]; in breast cancer by miR-124 [119,120], miR-30a [70], miR-497 [121], miR-1271 [122], and miR-203 [123,124]; in gastric cancer by miR-33a [125]; in lung cancer by miR-218 [126]; in clear cell renal cell carcinoma by miR-1 [127]; in osteosarcoma by miR-124 [128]; and in gingival fibroblasts by miR-200b [129]. Similarly to SNAIL, miRNAs–SLUG action regulates EMT in cancer progression, as well as different processes, such as the modulation of cancer stem cells’ activity. miR-204 binds to the 3′UTR regions of both *SLUG* and *SOX4* to suppress osteosarcoma cancer stem cells [117], whereas the loss of miR-124 enhances the stem-like traits of glioma cells [130]. The miRNAs–SLUG axis is also important in other biological processes, such as for example in traumatic heterotopic ossification. miR-630 inhibits endothelial–mesenchymal transition by targeting SLUG [131]. The regulation of SLUG expression by miRNAs is summarized in Table 3.

## 4. LncRNA, CircRNAs, and their Relationship to SNAIL and Targeting MicroRNAs

Besides miRNAs, an interesting mechanism of action in the regulation of SNAIL or SLUG expression is also described for long non-coding RNAs (lncRNA). The may act as sponges for miRNAs targeting SNAIL. LncRNA MALAT1 (metastasis associated lung adenocarcinoma transcript 1) acts as a competing endogenous RNA (ceRNA) by sponging miR-22 to promote melanoma growth and metastasis [83]. MALAT1 turned out to be a regulator of not only miR-22, but also miR-1-3p expression. In that way, it inhibits migration, invasion, and EMT, which leads to the increased expression of E-cadherin and decreased expression of vimentin, SLUG, and SNAIL [132]. Another interesting feature of MALAT1 is the modulation of cancer stem cells’ (CSC) activity by regulation of the miR-1/SLUG axis in nasopharyngeal carcinoma [133]. In gastric cancer, miR-22 is also regulated by lncRNA H19 with effects on metastasis via the miR-22-3p/SNAIL axis [134]. Another example in gastric cancer is lncRNA SNHG7 (small nucleolar RNA host gene 7), which directly binds to miR-34a and suppresses the miR-34a–SNAIL–EMT axis, which regulates gastric cancer cell migration and invasion [135].

SLUG level can also be regulated by other lncRNAs. For example, lncRNA GAPLINC (gastric adenocarcinoma associated) promotes the invasion of colorectal cancer by binding to PSF/NONO (probable DNA replication complex GINS protein PSF/non-POU domain-containing octamer-binding protein) and partly by stimulating the expression of SLUG [136]. lncRNA CAR10 directly binds two miRNAs: miR-30 and miR-203 and hence regulates the expression of both SNAIL and SLUG. In that way, it induces EMT and promotes lung adenocarcinoma metastasis [137]. In that cancer type, another example is lncRNA HCP5 acting as a sponge for miR-203 [138]. miR-203 interacts also with lncRNA UCA1 in hepatocellular carcinoma, and in that way, SLUG expression is regulated in tumor progression [139]. In that cancer type, lncRNA–AB209371 binds to hsa-miR199a-5p and weakens the inhibitory effect of hsa-miR199a-5p on SNAIL expression to promote EMT [140]. In breast cancer, lncRNA TINCR (terminal differentiation-induced ncRNA) targets miR-125b, and in that way regulates SNAIL and EMT [85].

LncRNAs may regulate the SNAIL level not only by miRNAs, but also epigenetically. LncRNA SATB2-AS1 (the antisense transcript of *SAT2B*—special AT-rich sequence-binding protein 2) mediates the epigenetic regulation of SNAIL expression in colorectal cancer progression. SATB2-AS1 recruits p300, whose acetylation of H3K27 and H3K9 at the *SATB2* promoter and subsequently the elevated SATB2 recruits HDAC1 to the *SNAIL* promoter to repress its transcription [141].

The interaction of lncRNAs with SNAIL is also possible. lncRNA NEAT1 (nuclear enriched abundant transcript 1) epigenetically suppresses E-cadherin expression in osteosarcoma cells by association with the G9a–DNMT1 (DNA methyltransferase 1)—SNAIL complex [142].

lncRNAs may also regulate the level of transcription factor by increasing their stability. For example, lncRNA SNHG15 impedes SLUG ubiquitination and its proteasomal degradation by interaction with the zinc finger domain of SLUG [143].

Besides lncRNAs, circular RNAs (circRNAs) were also described as SNAIL regulators. In hepatocellular carcinoma, circ-ZNF652 could physically interact with miR-203 and miR-502-5p to increase the expression of SNAIL. circ-ZNF652 was identified as a novel driver of EMT [96]. Similarly, in melanoma, circRNA_0084043 promotes progression via the miR-153-3p/SNAIL axis [144]. In urothelial carcinoma, circRNA PRMT5 acts as a sponge for miR-30c, which affects the SNAIL/E-cadherin pathway and thereby induces EMT [145]. circRNAs may be also implicated in the regulation of SLUG level. For example, circRNA-000284 can positively regulate the SLUG level in cervical cancer by sponging miR-506, which directly binds to *SLUG* 3′UTR [146].

The indirect regulation of SNAIL level by several mediators is also possible. Circular RNA hsa_circ_0008305 (circPTK2) inhibits TGF-β-induced EMT in non-small cell lung cancer by direct binding to miR-429/miR-200b-3p, which act as direct regulators of TIF1γ (transcriptional intermediary factor 1 γ), resulting in diminished SNAIL expression [147]. CircPIP5K1A induces non-small cell lung cancer progression by the regulation of miR-600/HIF-1α (hypoxia-inducible factor 1-alpha), which results in the upregulation of EMT-related factors, such as SNAIL [148]. Circ_0026344 promotes colorectal carcinoma invasion by targeting miR-183, which increases EMT and upregulates mesenchymal markers and SNAIL [149].

**Figure 3:**
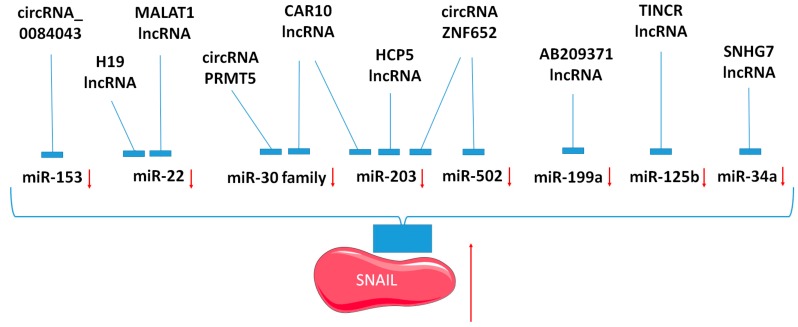
Role of long non-coding RNAs and circular RNAs as sponges for microRNAs in the regulation of SNAIL expression in tumors.

To summarize, SNAIL is regulated by signaling networks involving plenty of miRNAs, long non-coding RNAs, and circular RNAs (Table 4). lncRNAs and circRNAs usually act as sponges for miRNAs targeting SNAIL (Figure 3). This mechanism may be responsible for the regulation of tumor progression.

## 5. SNAIL Regulation of Non-Coding RNAs

MiRNAs were presented as regulators of SNAIL expression. On the other hand, there are several cases describing SNAIL as a regulator of miRNA level with implications to epithelial tumor progression and the role of EMT in this process. MiRNAs may be regulated either indirectly or by the direct binding of SNAIL to E-box sequences in miRNA promoters or regulatory sequences. 

For example, in breast cancer cells, SNAIL directly suppresses miR-182 [88] and miR-203 [86]. In head and neck cancers, SNAIL binds to the miR-493 promoter [150]. SNAIL also significantly represses the miR-145 promoter. miR-145 plays a role in antagonizing SNAIL-mediated stemness in colorectal cancer [151]. In gastric cancer, SNAIL binds to the putative promoter of miR-375 [152]. SNAIL directly activates the transcription of miR-21 to produce exosomes abundant in miR-21, which promotes the M2-like polarization of tumor-associated macrophages [153].

In non-epithelial tumor types, such as glioma, SNAIL suppresses miR-128b expression by direct binding to the miR-128b-specific promoter motif; then, miR-128 and SP1 regulate tumor progression [154]. A similar direct mechanism was demonstrated for miR-128-2 in mammary epithelial cells. The loss of SNAIL-regulated miR-128-2 targets multiple stem cell factors to promote the oncogenic transformation of mammary epithelial cells [155]. The SNAIL/miR-128 axis regulates the growth, invasion, metastasis, and EMT of gastric cancer. miR-128 targets directly Bmi11, and it can reverse EMT induced by Bmi-1 via the PI3K/AKT pathway, whereas SNAIL curbs the expression of miR-128, and then down-regulated miR-128 promotes the expression of Bmi-1 [156]. The loss of SNAIL was also shown to inhibit cellular growth and metabolism through the miR-128-mediated signaling pathway in prostate cancer cells [157].

Interestingly, SNAIL may also exert its effects by epigenetic modifications. SNAIL is involved in CpG DNA methylation of the miR-200f loci, which is essential for maintenance of the mesenchymal phenotype. In the MDCK (Madin-Darby canine kidney) epithelial kidney cells model, it has been shown that ZEB1 and SNAIL engage miR-200f transcriptional and epigenetic regulation during EMT [158]. Regulation of the miR-200 family by SNAIL also plays a role in vasculogenesis and may be significant both in malignant cancer and in early developing embryos [159].

SNAIL overexpression increases the level of miR-125b through the SNAIL-activated Wnt/β-catenin/TCF4 (transcription factor 4) axis. This mechanism was described for SNAIL-induced stem cell propagation [160]. Another example of SNAIL action in cancer stem cells is signaling axis involving SNAIL, miR-146a, and Numb in regulation of the switch between symmetric and asymmetric cell division in colorectal cancer stem cells [161].

As indicated previously, SNAIL is a regulator of not only EMT and cancer stem cells, but also of myogenic differentiation. In rhabdomyosarcoma, SNAIL regulates the expression of myogenic-associated miRNAs, such as miR-1, miR-206, and miR-378 [14]. What is more, the SNAIL/miR-199a-5p axis promotes the differentiation of fibroblasts into myofibroblasts by the induction of endothelial–mesenchymal transition [162].

There are also examples of interaction among lncRNAs, miRNAs, and SNAIL. SNAIL binds to the promoter of lncRNA PCA3 and activates its expression. Then, lncRNA PCA3 inhibits the translation of PRKD3 (serine/threonine-protein kinase D3) protein via competitive miR-1261 sponging and in that way promotes the invasion of prostate cancer cells [163].

SNAIL’s role has been also described in controlling telomere transcription and integrity, which may be significant features of cancer stem cells, since telomere maintenance is essential for stemness. SNAIL turned out to be a negative regulator of lncRNA that controls telomere integrity, which is called telomeric repeat-containing RNA (TERRA). What is more, TERRA can also affect the transcription of some genes induced during EMT [164].

SNAIL may also not only regulate the level of lncRNAs, but it may also interact with them to modify the chromatin. lncRNA HOTAIR (HOX Transcript Antisense Intergenic RNA) mediates a physical interaction between SNAIL and EZH2 (enhancer of zeste homolog 2), which is an enzymatic subunit of the polycomb-repressive complex 2. In that way, SNAIL recruits EZH2 to specific genomic sites during EMT [165].

SNAIL may also regulate circRNAs. For example, SNAIL targets the E-box motif on the promoter of circ-ZNF652 to increase its expression [96].

Besides SNAIL, similar mechanisms of binding to miRNA promoters were also described for SLUG. In colorectal cancer, SLUG binds to miR-145 promoter and represses it to modulate 5-fluorouracil sensitivity [166]. In lung cancer cells, SLUG binds directly to the E-box in the promoter of miR-137 and acts as an activator, which promotes cancer invasion and progression by directly suppressing TFAP2C (transcription factor AP-2 gamma) [167]. In prostate cancer, SLUG is a direct repressor of miR-1 and miR-200 transcription [168]. In breast cancer cells, SLUG directly binds to miR-203 promoter, downregulating its expression [124]. SLUG-upregulated miR-221 promotes breast cancer progression through suppressing E-cadherin expression, which indicates that miR-221 is an additional blocker of E-cadherin besides SNAIL and SLUG [169].

Sometimes, both SNAIL and SLUG collaborate on EMT and tumor metastasis through miRNAs. In oral tongue squamous cell carcinoma, those transcription factors act through the miR-101-mediated EZH2 axis [170]. miR-101 functions as a tumor suppressor by directly targeting *ZEB1* (zinc finger E-Box binding homeobox 1) in various cancers, including colorectal cancer [171].

**Figure 4:**
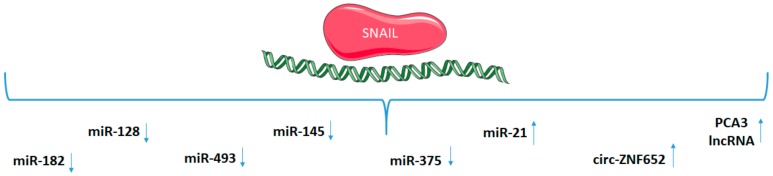
MiRNAs, long non-coding RNAs (lncRNAs), and circular RNAs regulated directly by SNAIL transcription factor.

To summarize, SNAIL and SLUG may be direct or indirect regulators of miRNAs, lncRNAs, and circRNAs (Table 5). There are several examples of direct binding SNAIL to promoters or regulatory sequences of non-coding RNAs (Figure 4). Subsequently, those RNAs target plenty of genes to regulate tumor progression.

## 6. Multi-Component Feedback Loops and Multi-Component Signaling Networks

The literature also describes several examples of multi-component feedback loops and multi-component signaling networks involving the SNAIL transcription factor and non-coding RNAs.

**Figure 5:**
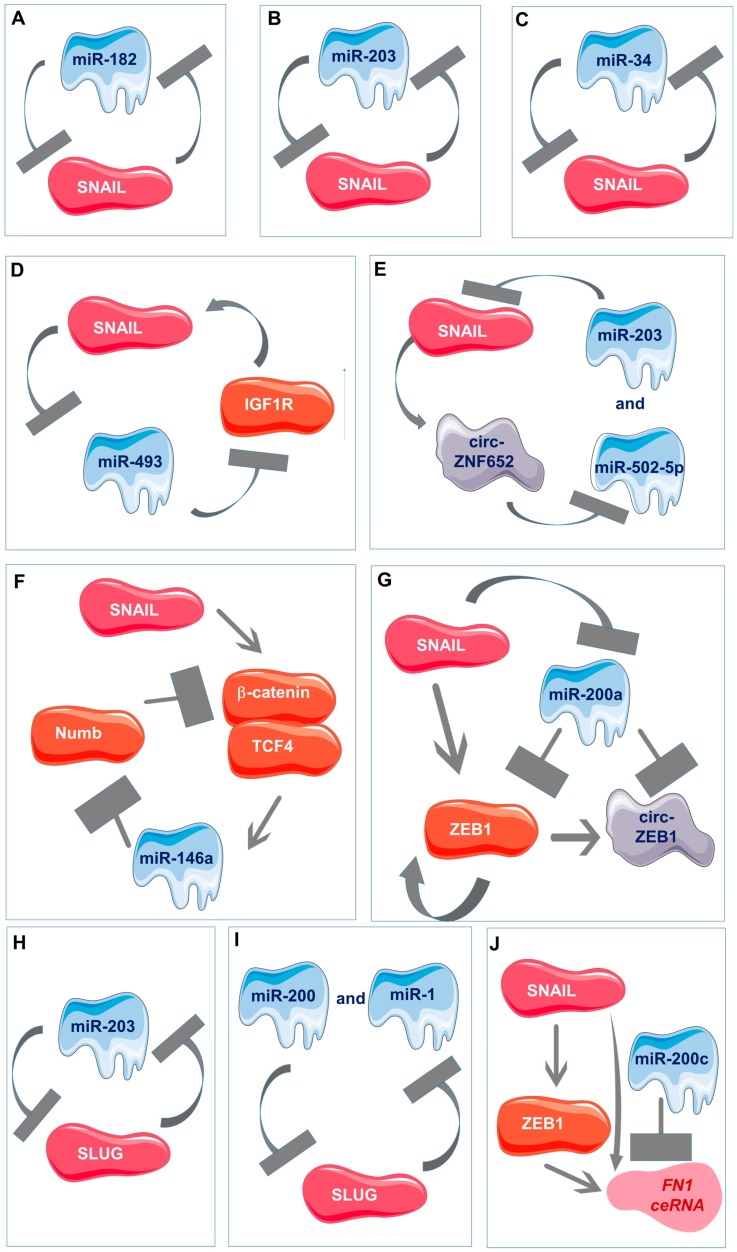
Multi-component feedback loops and multi-component signaling networks involving SNAIL and non-coding RNAs. (**A**) Negative regulation between SNAIL and miR-182. (**B**) Negative regulation between SNAIL and miR-203. (**C**) Negative regulation between SNAIL and miR-34. (**D**) Feedback loop between SNAIL, miR-493, and IGF1R. (**E**) Feedback loop between SNAIL, circ-ZNF652, miR-203, and miR-502-5p. (**F**) Signaling pathway involving SNAIL, β-catenin, miR-146a, and Numb. (**G**) Signaling pathway involving SNAIL, ZEB1, miR-200a, and circ-ZEB1. (**H**) Negative regulation between SLUG and miR-203. (**I**) Negative regulation among SLUG, miR-1, and miR-200. (**J**) Signaling pathway involving SNAIL, ZEB1, miR-200c, and *FN1* ceRNA.

Selected different multi-component feedback loops and multi-component signaling networks are presented in Figure 5.

An interesting example is miR-182, which is directly suppressed by SNAIL in breast cancer cells, which can also target its suppressor (Figure 5A). This mechanism regulates an epithelial-like phenotype in vitro and enhances macrometastases in vivo [88].

Similarly in breast cancer, miR-203 forms also a double-negative miR-203/SNAIL feedback loop, as SNAIL reduces the activity of the miR-203 promoter (Figure 5B) [86].

Moreover, miR-34 and SNAIL form a double-negative feedback loop (Figure 5C) [73] that may feed-forward regulate ZNF281/ZBP99 to promote EMT, which has implications for human colon and breast cancer [172]. The expression of ZNF281 (zinc finger protein 281) is induced by SNAIL and inhibited by miR-34a, which mediates the repression of ZNF281 by the p53 tumor suppressor. The deregulation of this circuitry by mutational and epigenetic alterations in the p53/miR-34a axis promotes colorectal cancer metastasis [173].

In head and neck cancers, SNAIL binds to miR-493 promoter to repress it, and subsequently, miR-493 forms a negative feedback loop with the insulin-like growth factor 1 receptor pathway to block tumorigenesis (Figure 5D) [150].

Besides miRNAs, SNAIL may also form feedback loops with circular RNAs. SNAIL upregulates circ-ZNF652 by binding to the E-box motif on the promoter. Subsequently, circ-ZNF652 acts a sponge for miR-203 and miR-502-5p, which target *SNAIL* 3′UTR (Figure 5E) [96].

In cancer stem cells, SNAIL forms a feedback circuit to maintain Wnt activity. SNAIL induces miR-146a expression through the β-catenin-TCF4 complex, and subsequently, miR-146a targets Numb to stabilize β-catenin (Figure 5F) [161].

An interesting example is also SNAIL action in ZEB1 circuit in melanoma cells. SNAIL is considered as an external signal that transcriptionally regulates the ZEB1/miR-200a/cicrZEB1 axis. circZEB1, generated from the ZEB1 gene, contains a binding site for mir200a, which is a post-transcriptional regulator of ZEB1 (Figure 5G) [174].

SLUG and microRNAs may also form regulatory loops. In breast cancer cells, SLUG and miR-203 form a double-negative feedback loop and SLUG directly binds to miR-203 promoter, downregulating its expression in metastatic breast cancer cells (Figure 5H) [124]. Furthermore, SLUG and miR-1/miR-200 act in a self-reinforcing regulatory loop, which results in EMT amplification (Figure 5I) [168]. 

What is also interesting is that sometimes, gene transcripts may also act as a competitive endogenous RNA (ceRNA) to regulate biological processes. *FN1* (fibronectin 1) acts as a ceRNA for miR-200c in the canonical SNAIL-ZEB-miR200 pathway in breast cancer cells (Figure 5J), whereas *TGFBI* (transforming growth factor-beta-induced) is a transcript that is highly induced during EMT in lung cancer cells, which acts as the ceRNA for miR-21 to modulate EMT [175].

## 7. Conclusions

SNAIL participates in many physiological and pathological processes, including embryonic development and cancer metastasis. Therefore, the identification of its crosstalk with non-coding RNAs can help in understanding the complex signaling networks that drive tumor progression. Unraveling these signaling networks may help generate new types of cancer therapeutics. miRNAs and other non-coding RNAs play key roles in tumor progression or suppression. One miRNA may target multiple genes besides *SNAIL*. Therapies targeting miRNA may enable the regulation of more than one signaling pathway. An interesting example of miRNA (described in this review) therapeutics is a drug based on miR-34a mimics, which has been already enrolled in clinical trials [176]. The identification of miRNA downstream and upstream of SNAIL may create novel possibilities for biomarker determination during cancer progression, which may lead to improvements in prognosis and therapy. As those miRNAs usually regulate epithelial to mesenchymal transition, their identification may help to distinguish different stages of tumor development, as well as benign and malignant tumors. For the identification of novel biomarkers, the next step is verification of whether miRNA candidates can be secreted from tumor to blood vessels.

## Figures and Tables

**Table 1:** MicroRNAs regulating SNAIL.

MicroRNA	Cancer/Cell Type	References
miR-22	lung cancer	[81]
	bladder cancer	[82]
	melanoma	[83]
	gastric cancer	[84]
miR-30 family	non-small cell lung carcinoma	[62]
	breast cancer	[63]
	pancreatic cancer	[64]
	melanoma	[65]
	esophageal squamous cell carcinoma	[66]
	rhabdomyosarcoma	[14]
	hepatocytes	[67,68]
miR-34	colon carcinoma	[71]
	breast carcinoma	[71]
	lung carcinoma	[71]
	ovarian cancer	[72]
	pancreatic cancer	[74]
miR-122	hepatocellular carcinoma	[95]
miR-125b	breast cancer	[85]
miR-130b	diabetic nephropathy	[100]
miR-133	fibroblasts	[99]
miR-137	ovarian cancer	[72]
miR-153	laryngeal squamous cell carcinoma	[75]
	melanoma	[76]
	esophageal squamous cell carcinoma	[77]
	gastric cancer	[78]
	hepatocellular carcinoma	[79]
	pancreatic ductal adenocarcinoma	[80]
miR-182	breast cancer	[88]
miR-199a	lung cancer	[91]
	papillary thyroid carcinoma	[93]
miR-203	breast cancer	[86]
miR-204	gastric cancer	[90]
miR-211-5p	renal cancer	[98]
miR-363	ovarian cancer	[94]
miR-410-3p	breast cancer	[87]
miR-486-5p	prostate cancer	[97]
miR-491-5p	gastric cancer	[89]
miR-502-5p	hepatocellular carcinoma	[96]
miR-940	lung cancer	[92]

**Table 2:** Signaling pathways involving microRNAs that regulate SNAIL.

MicroRNA	Regulated Pathway and Genes	Mechanism of SNAIL Regulation	Cancer/Cell Type	References
miR-9	NF-κB1	SNAIL expression	melanoma	[108]
miR-101	CXCL12-mediated AKT	SNAIL localization	thyroid carcinoma	[104]
miR-126	PI3K-AKT	SNAIL localization	lung cancer	[102]
miR-148a	MET/AKT/GSK-3β	SNAIL localization and degradation	hepatoma cells	[101]
miR-181b-3p	YWHAG protein	SNAIL stabilization	breast cancer	[106]
miR-215	PI3K-AKT	SNAIL localization	papillary thyroid cancer	[103]
miR-1306-3p	FBXL5	Suppression of SNAIL degradation	hepatocellular carcinoma	[105]
miR-5003-3p	MDM2, E-cadherin	SNAIL stabilization	breast cancer	[107]

**Table 3:** MicroRNAs regulating SLUG.

MicroRNA	Cancer/Cell Type	References
miR-1	lung cancer	[118]
miR-30a	breast cancer	[70]
miR-33a	gastric cancer	[125]
miR-124	breast cancer	[119,120]
	osteosarcoma	[128]
	glioma	[130]
miR-200b	gingival fibroblasts	[129]
miR-203	glioblastoma	[117]
	breast cancer	[123,124]
miR-204	oral squamous cell carcinoma	[116]
miR-218	lung cancer	[126]
miR-497	breast cancer	[121]
miR-630	dermal microvascular endothelial cells	[131]
miR-1271	breast cancer	[122]

**Table 4:** Long non-coding RNAs regulating SNAIL and SLUG.

LncRNA/CircRNA	Regulated MicroRNAs	Regulated Factors	Cancer	References
lncRNA MALAT1	miR-22	SNAIL	melanoma	[83]
	miR-22 and miR-1-3p	E-cadherin, vimentin, SLUG and SNAIL	prostate cancer	[132]
	miR-1	SLUG	nasopharyngeal carcinoma	[133]
lncRNA H19	miR-22-3p	SNAIL	gastric cancer	[134]
lncRNA SNHG7	miR-34a	SNAIL	gastric cancer	[135]
lncRNA CAR10	miR-30 and miR-203	SNAIL and SLUG	lung adenocarcinoma	[137]
lncRNA HCP5	miR-203	SNAIL	lung adenocarcinoma	[138]
lncRNA UCA1	miR-203	SLUG	hepatocellular carcinoma	[139]
lncRNA AB209371	miR199a-5p	SNAIL	hepatocellular carcinoma	[140]
lncRNA TINCR	miR-125b	SNAIL	breast cancer	[85]
lncRNA SATB2-AS1	-	SNAIL (epigenetic regulation involving SATB2)	colorectal cancer	[141]
lncRNA NEAT1	-	E-cadherin by association with G9a-DNMT1-SNAIL complex	osteosarcoma cells	[142]
lncRNA SNHG15	-	SNAIL (ubiquitination by interaction with zinc finger domain)	colon cancer	[143]
lncRNA GAPLINC	-	SLUG (by binding to PSF/NONO)	colorectal cancer	[136]
circ-ZNF652	miR-203 and miR-502-5p	SNAIL	hepatocellular carcinoma	[96]
circRNA_0084043	miR-153-3p	SNAIL	melanoma	[144]
circRNA PRMT5	miR-30c	SNAIL	urothelial carcinoma	[145]
circRNA-000284	miR-506	SLUG	cervical cancer	[146]
hsa_circ_0008305 (circPTK2)	miR-429 and miR-200b-3p	SNAIL (indirectly by TIF1γ)	non-small cell lung cancer	[147]
circPIP5K1A	miR-600	SNAIL (indirectly by HIF-1α)	non-small cell lung cancer	[148]
circ_0026344	miR-183	SNAIL (indirectly)	colorectal cancer	[149]

**Table 5:** Non-coding RNAs regulated by SNAIL and SLUG.

Non-Coding RNA	Mechanism	Cancer/Cell Type	References
miR-1	SLUG binding to promoter	prostate cancer	[168]
	regulation by SNAIL (unknown mechanism)	rhabdomyosarcoma	[14]
miR-21	SNAIL binding to promoter	head and neck cancer	[153]
miR-101	transcriptional control by SNAIL and SLUG	squamous cell carcinoma	[170]
miR-125b	SNAIL-activated Wnt/β-catenin/TCF4 axis	breast cancer stem cells	[160]
miR-128	SNAIL binding to promoter	glioma	[154]
		prostate cancer	[157]
		gastric cancer	[156]
miR-137	SLUG binding to promoter	lung cancer	[167]
miR-145	SNAIL binding to promoter	colorectal cancer	[151]
	SLUG binding to promoter	colorectal cancer	[166]
miR-146a	SNAIL-induced β-catenin-TCF4 complex	colorectal cancer stem cells	[161]
miR-182	SNAIL binding to promoter	breast cancer	[88]
miR-200	SNAIL involved in CpG DNA methylation	human kidney cells	[158]
	SLUG binding to promoter	prostate cancer	[168]
miR-203	SNAIL binding to promoter	breast cancer	[86]
	SLUG binding to promoter	breast cancer	[124]
miR-206	regulation by SNAIL (unknown mechanism)	rhabdomyosarcoma	[14]
miR-221	transcriptional control by SLUG	breast cancer	[169]
miR-375	SNAIL binding to promoter	gastric cancer	[152]
miR-378	regulation by SNAIL (unknown mechanism)	rhabdomyosarcoma	[14]
miR-493	SNAIL binding to promoter	head and neck cancer	[150]
lncRNA PCA3	SNAIL binding to promoter	prostate cancer	[163]
lncRNA TERRA	transcriptional control by SNAIL	mesenchymal stem cells and mammary cells	[164]
lncRNA HOTAIR	interaction of SNAIL with HOTAIR and EZH2	hepatocytes	[165]
circ-ZNF652	SNAIL binding to promoter	hepatocellular carcinoma	[96]
